# Supplementary material for: Transcriptomic analysis of mesocarp tissue during fruit development of the oil palm revealed specific isozymes related to starch metabolism that control oil yield
Source: Front Plant Sci. 2023 Jul 24;14:1220237. doi: 10.3389/fpls.2023.1220237 (PMC10405827; doi:10.3389/fpls.2023.1220237)
Supplement: Supplementary file 1 [file DataSheet_1.pdf]

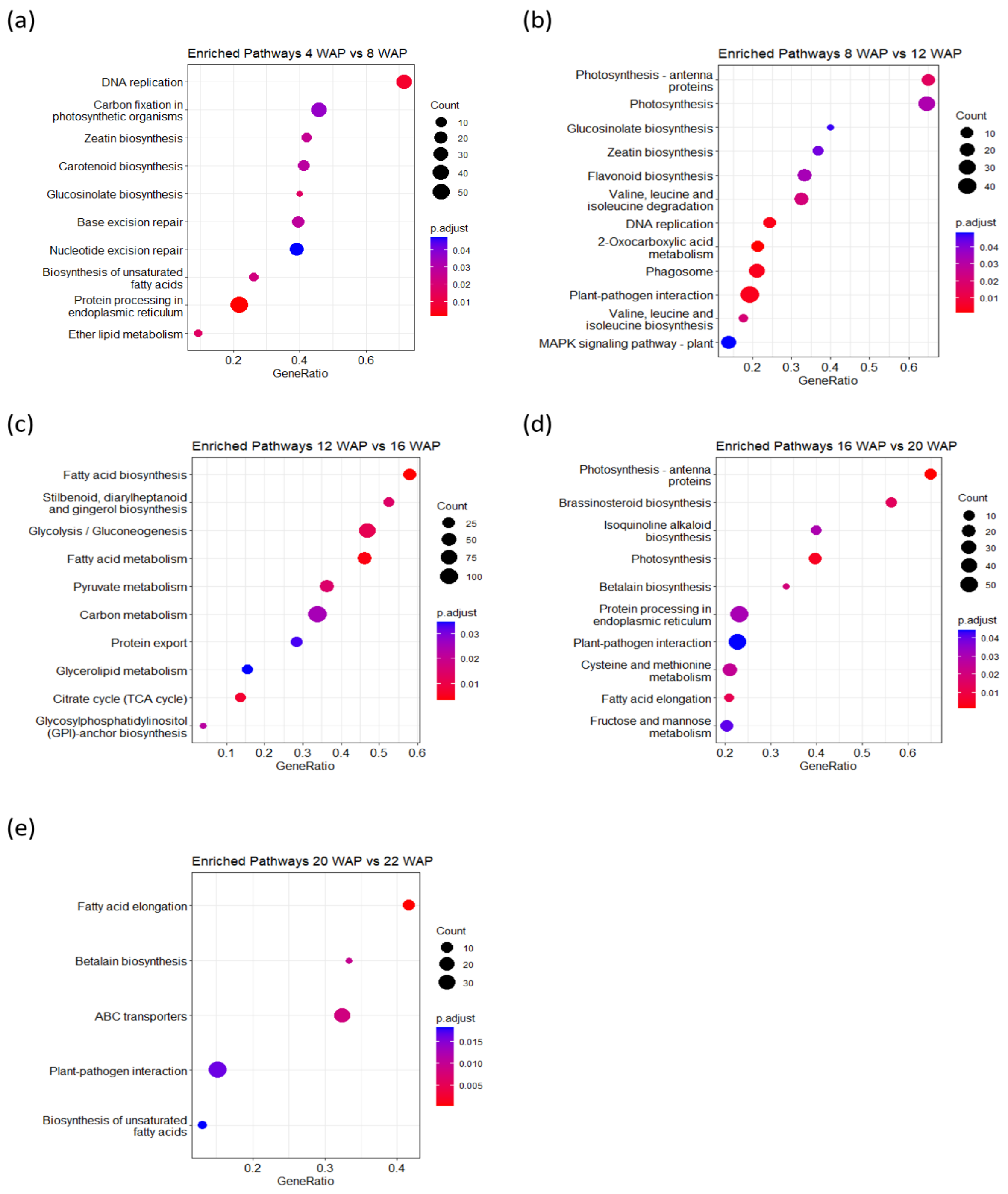

Figure S1. KEGG enrichment analysis of DEGs in oil palm fruit at different developmental stages. (a-e) Represents enrichment results in each pairwise comparison during fruit development, respectively. The vertical axis indicated the corresponding rich factor for each pathway, the horizontal axis represented the pathway name, and the color of the dots reflected the p-value. The size of the dots reflects the number of differentially enriched genes.
