# Supplementary material for: Transcriptomic analysis of mesocarp tissue during fruit development of the oil palm revealed specific isozymes related to starch metabolism that control oil yield
Source: Front Plant Sci. 2023 Jul 24;14:1220237. doi: 10.3389/fpls.2023.1220237 (PMC10405827; doi:10.3389/fpls.2023.1220237)
Supplement: Supplementary file 3 [file DataSheet_3.pdf]

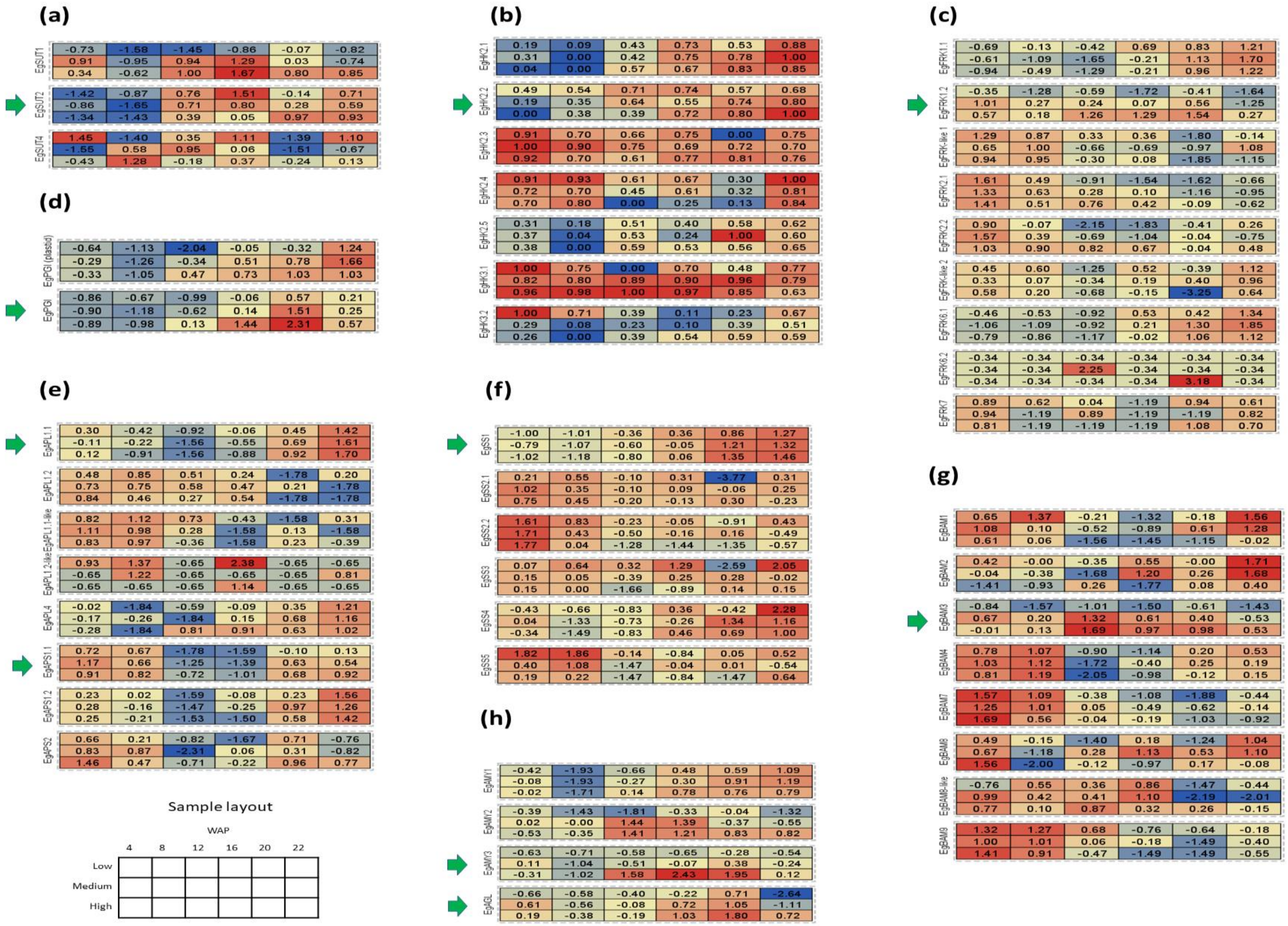

Figure S3. Detailed heatmap analysis of specific isoform related to yield in starch metabolism pathway. The green arrow show highly correlated genes expression with oil yield group. Color and value corresponds to per-gene z-score that is computed from normalized values of log2 (CPM+1). Blue shows lower expression and red shows higher expression value.
