# Supplementary material for: Transcriptomic analysis of mesocarp tissue during fruit development of the oil palm revealed specific isozymes related to starch metabolism that control oil yield
Source: Front Plant Sci. 2023 Jul 24;14:1220237. doi: 10.3389/fpls.2023.1220237 (PMC10405827; doi:10.3389/fpls.2023.1220237)
Supplement: Supplementary file 4 [file DataSheet_4.pdf]

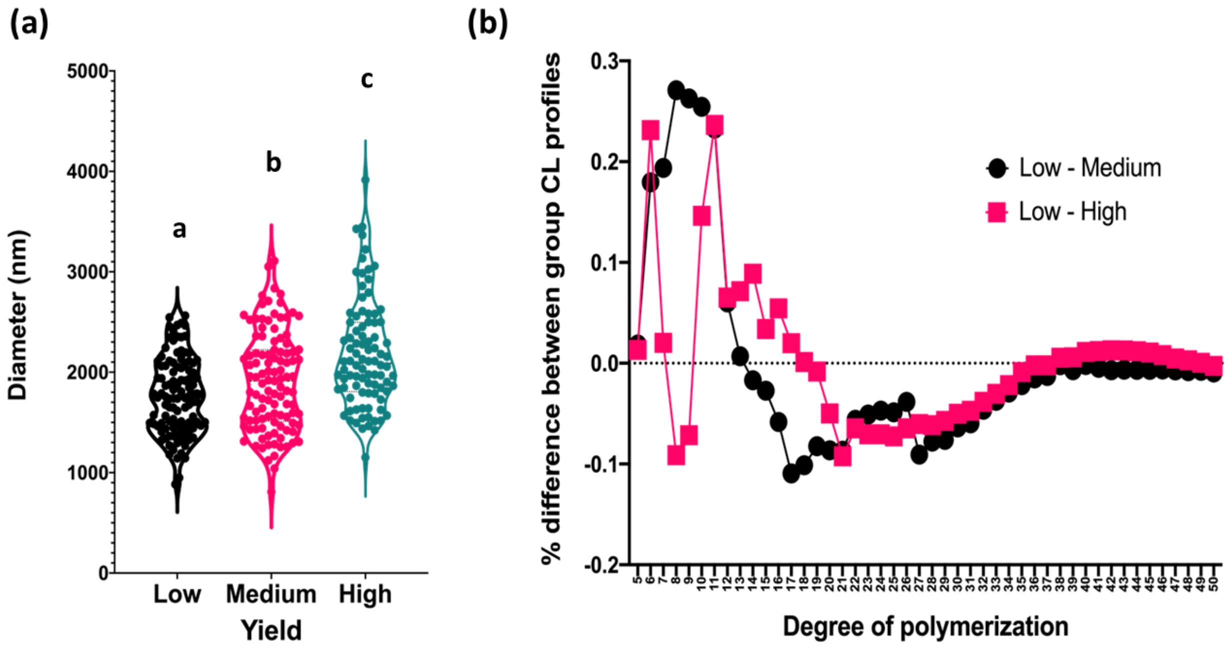

Figure S4. Starch granule size distribution and differential chain length distribution at 22 WAP. (a) Starch granule size distribution for different yield categories at 22WAP. Each group represents 100 starch granules, and a letter indicates statistical significance between samples (one-way ANOVA; $p < 0.05$; Tukey's test) (Apriyanto et al., 2022b). (b) Difference plots corresponding to chain length distribution profiles between oil yield groups. Mean profiles of samples wer e used to draw difference plots (Apriyanto et al., 2022b).
