# Supplementary material for: Transcriptomic analysis of mesocarp tissue during fruit development of the oil palm revealed specific isozymes related to starch metabolism that control oil yield
Source: Front Plant Sci. 2023 Jul 24;14:1220237. doi: 10.3389/fpls.2023.1220237 (PMC10405827; doi:10.3389/fpls.2023.1220237)
Supplement: Supplementary file 5 [file DataSheet_5.pdf]

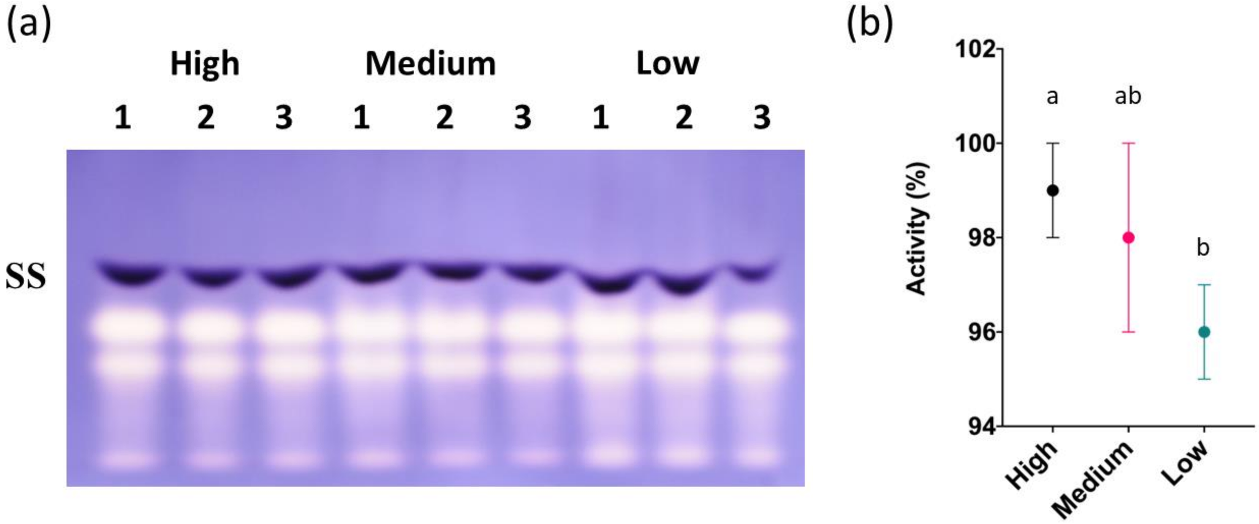

Figure S5. Activity staining of (a) soluble starch synthase (SS). Each lane was loaded with 50 µg of proteins extracted from mesocarp tissue. Gel was stained using Lugol solution. The samples used were 22 weeks after pollination (WAP). (b) Enzymes activities with different oil yield at 22 WAP. Each sample represents three biological replicates. The intensity of the obtained activity bands was quantified using the software GelQuant.Net. Values are shown as means ± standard deviation (SD), and a letter indicates statistical significance between samples (one-way ANOVA, $p < 0.05$; Tukey's test).
