# Supplementary material for: Transcriptomic analysis of mesocarp tissue during fruit development of the oil palm revealed specific isozymes related to starch metabolism that control oil yield
Source: Front Plant Sci. 2023 Jul 24;14:1220237. doi: 10.3389/fpls.2023.1220237 (PMC10405827; doi:10.3389/fpls.2023.1220237)
Supplement: Supplementary file 6 [file DataSheet_6.pdf]

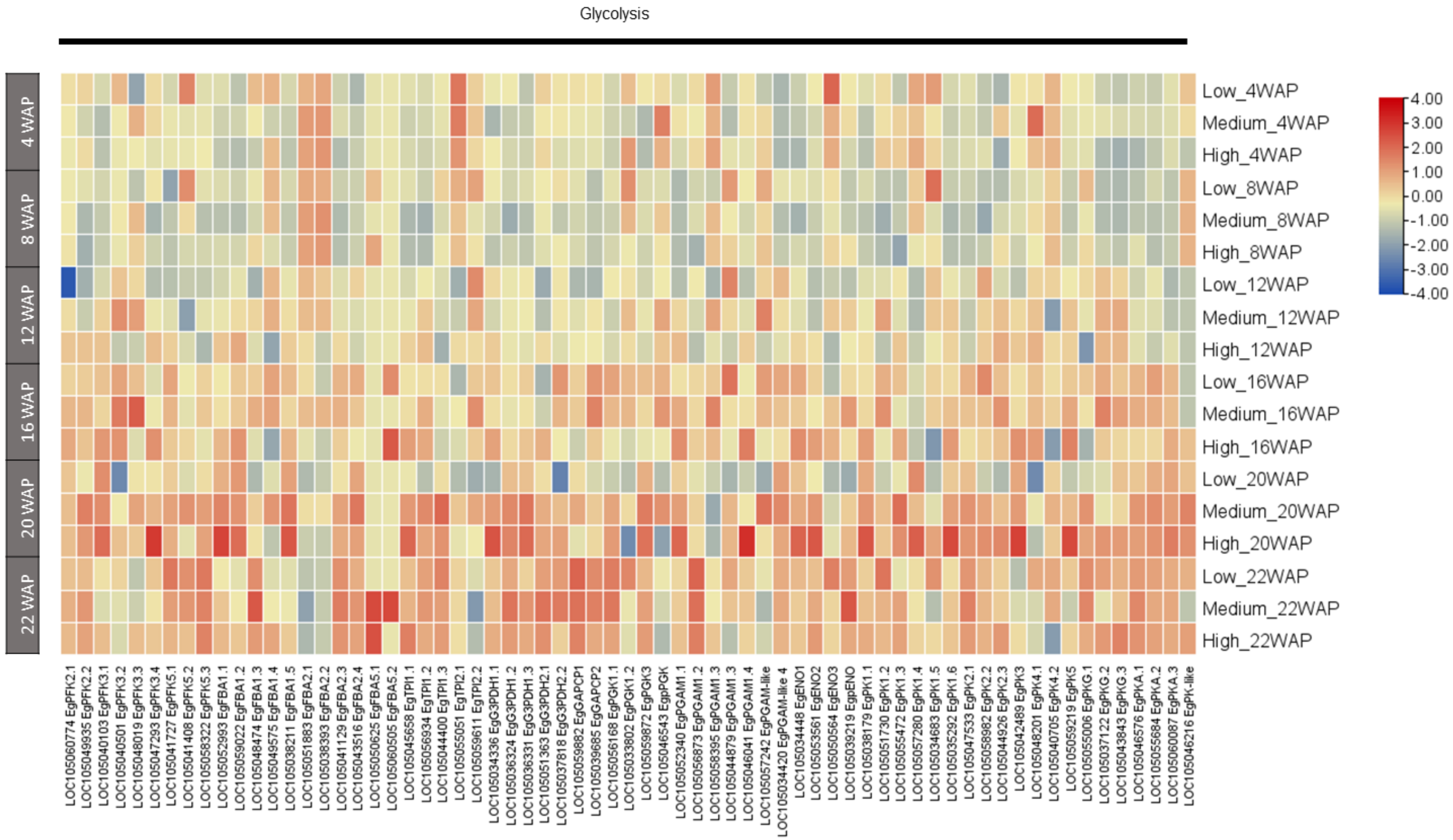


**Figure S6.** Heatmap analysis of glycolysis genes during fruit development. Comparison of gene expression with altered oil yield at different developmental stages. Color corresponds to per-gene z-score that is computed from normalized values of log2 (CPM+1).
