# Supplementary material for: Transcriptomic analysis of mesocarp tissue during fruit development of the oil palm revealed specific isozymes related to starch metabolism that control oil yield
Source: Front Plant Sci. 2023 Jul 24;14:1220237. doi: 10.3389/fpls.2023.1220237 (PMC10405827; doi:10.3389/fpls.2023.1220237)
Supplement: Supplementary file 7 [file DataSheet_7.pdf]

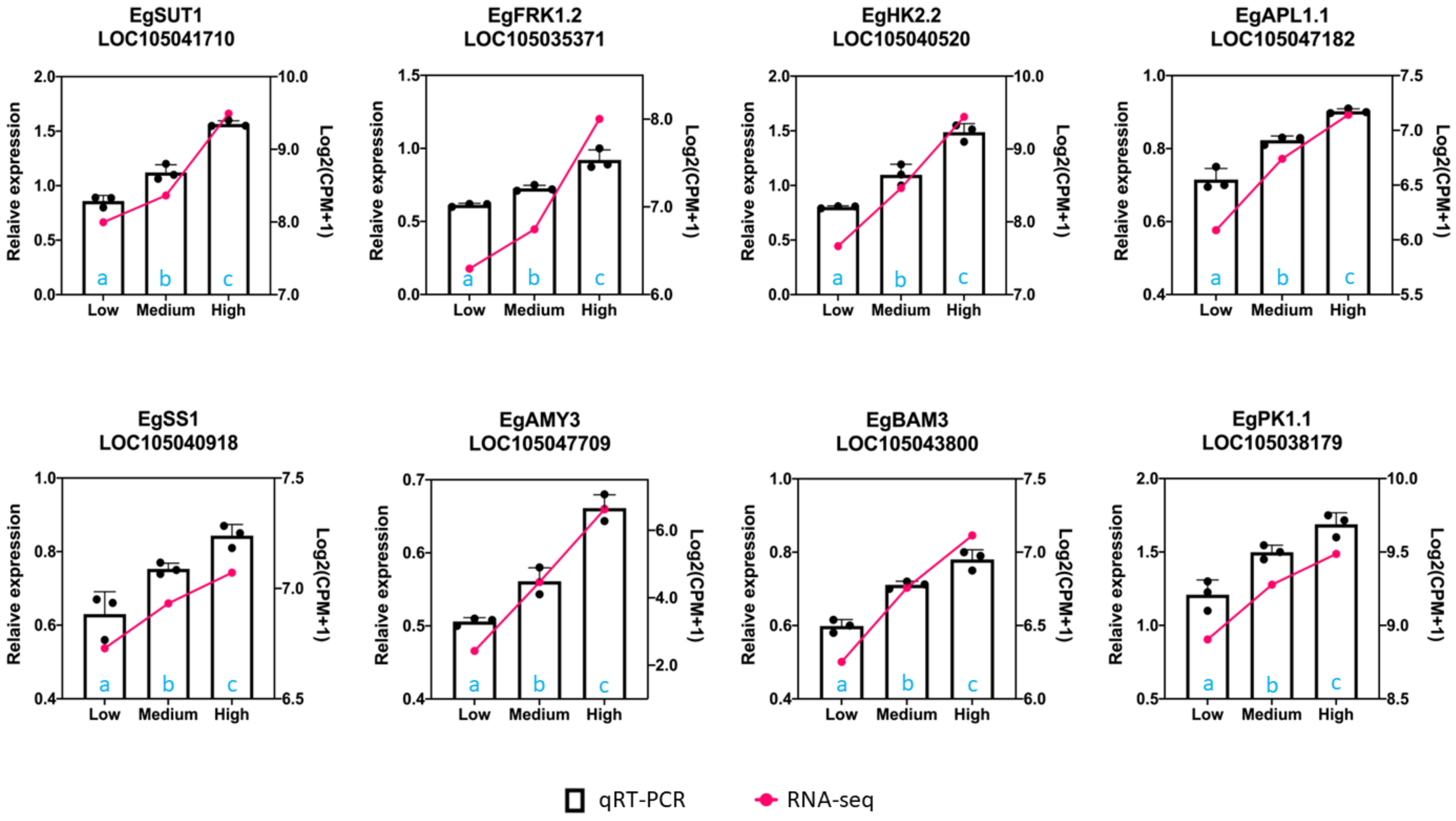

Figure S7. Validation of RNA-seq results by qRT-PCR for selected genes. Three biological replicates was used for validation. The gene expression was relative to the β-Actin (housekeeping gene). For statistical analyses, one-way ANOVA with Tukey post-hoc test was applied to each group ($n = 3$), letters indicate a significant difference between corresponding groups ($p < 0.001$).
