# Supplementary material for: Transcriptomic analysis of mesocarp tissue during fruit development of the oil palm revealed specific isozymes related to starch metabolism that control oil yield
Source: Front Plant Sci. 2023 Jul 24;14:1220237. doi: 10.3389/fpls.2023.1220237 (PMC10405827; doi:10.3389/fpls.2023.1220237)
Supplement: Supplementary file 8 [file DataSheet_8.pdf]

Table S1. List of starch metabolism genes investigated in this study. Note, genes were correlated to predicted main involved pathway. However, some of the genes are known to be involved in synthesis and degradation.

No	Function	Pathway	Gene	Chr	Position	Oil Palm Locus	Arabidopsis Locus
1	Starch Synthase 1	Synthesis	EgSS1	3	14965902-14997822	LOC105040918	AT5G24300
2	Starch Synthase 2	Synthesis	EgSS2.1	3	57555412-57565796	LOC105042381	AT3G01180
3	Starch Synthase 2	Synthesis	EgSS2.2	15	19368153-19379658	LOC105058547	AT3G01180
4	Starch Synthase 3	Synthesis	EgSS3	2	1580416-1621033	LOC105035295	AT1G11720
5	Starch Synthase 4	Synthesis	EgSS4	1	18936342-18957432	LOC105048676	AT4G18240
6	Starch Synthase 5	Synthesis	EgSS5	Un	17174-37334	LOC105035933	AT5G65685
7	Phosphoglucoisomerase	Synthesis	EgPGI	15	18912067-18931067	LOC105058489	AT4G24620
8	Phosphoglucoisomerase	Synthesis	EgPGI (Cyt)	1	59353842-59374007	LOC105060694	AT5G42740
9	Phosphoglucomutase 1	Synthesis	EgPGM1	11	14973755-14991294	LOC105054280	AT5G51820
10	Phosphoglucomutase 2	Synthesis	EgPGM2.1	3	40934272-40981969	LOC105042097	AT1G70730
11	Phosphoglucomutase 2	Synthesis	EgPGM2.2	7	12047241-12064834	LOC105048493	AT1G70730
12	AGPase large subunit 1	Synthesis	EgAPL1.1	6	33514039-33523776	LOC105047182	AT5G19220
13	AGPase large subunit 1	Synthesis	EgAPL1.2	Un	107603-121218	LOC105034020	AT5G19220
14	AGPase large subunit 1-like	Synthesis	EgAPL1.1-like	1	19185839-19210689	LOC105048507	
15	AGPase large subunit 1-like	Synthesis	EgAPL1.2-like	Un	91089-93909	LOC105059958	
16	AGPase large subunit 4	Synthesis	EgAPL4	2	65204124-65212882	LOC105040139	AT2G21590
17	AGPase small subunit 1	Synthesis	EgAPS1.1	Un	264328-271822	LOC105060488	AT5G48300
18	AGPase small subunit 1	Synthesis	EgAPS1.2	8	34541224-34549328	LOC105050621	AT5G48300
19	AGPase small subunit-like 2	Synthesis	EgAPS2	2	2717891-2726171	LOC105035803	AT1G05610
20	Granule-bound starch synthase 1	Synthesis	EgGBSS1	16	8821651-8827182	LOC105059288	AT1G32900
21	Granule-bound starch synthase 1b	Synthesis	EgGBSS1b	4	245058-250091	LOC105042450	
22	Granule-bound starch synthase 1-like	Synthesis	EgGBSS1-like	Un	1069-2665	LOC105037411	
23	MAR-binding filament-like protein 1	Synthesis	EgMFP1	3	2515104-2529539	LOC105040282	AT3G16000
24	Protein involved in starch initiation	Synthesis	EgPII1	Un	625093-650615	LOC105033456	AT4G32190

25	Protein targeting to starch 1	Synthesis	EgPTST1	3	22108143-22129869	LOC105041290	AT5G39790
26	Protein targeting to starch 2	Synthesis	EgPTST2	1	35599016-35622367	LOC105053977	AT1G27070
27	Protein targeting to starch 3	Synthesis	EgPTST3	4	27835790-27879666	LOC105042962	AT5G03420
28	Starch branching enzyme 1	Synthesis	EgBE1	1	49265563-49310595	LOC105058934	AT3G20440
29	Starch branching enzyme 2	Synthesis	EgBE2	12	20105720-20117193	LOC105055201	AT5G03650
30	Starch branching enzyme 3	Synthesis	EgBE3	7	10890859-10941698	LOC105048541	AT2G36390
31	Sucrose synthase 1 (SuSy1)	Synthesis	EgSuSy1	15	19984390-19992289	LOC105058592	AT5G20830
32	Sucrose synthase 1-like	Synthesis	EgSuSy1.1-like	10	22796192-22803424	LOC105053047	
33	Sucrose synthase 1-like	Synthesis	EgSuSy1.2-like	3	38083578-38090524	LOC105042002	
34	Sucrose synthase 2 (SuSy2)	Synthesis	EgSuSy2	13	24346695-24355705	LOC105056682	AT5G49190
35	Sucrose synthase 4 (SuSy4)	Synthesis	EgSuSy4	5	15730527-15746219	LOC105045149	AT3G43190
36	Sucrose synthase 7 (SuSy7)	Synthesis	EgSuSy7	12	21623272-21627625	LOC105055284	AT5G37180
37	Sucrose synthase 7-like	Synthesis	EgSuSy7-like	16	4777805-4782243	LOC105058897	
38	Invertase, beta-fructofuranosidase, 1	Synthesis	EgcINV1.1	16	17150268-17155617	LOC105059505	AT1G35580
39	Invertase, beta-fructofuranosidase, 1	Synthesis	EgcINV1.2	11	21474855-21478403	LOC105054034	AT1G35580
40	Invertase, beta-fructofuranosidase, insoluble isoenzyme 3	Synthesis	EgcWINV3.1	2	22294122-22298869	LOC105038262	AT1G62660
41	Invertase, beta-fructofuranosidase, insoluble isoenzyme 3	Synthesis	EgcWINV3.2	9	36916172-36920495	LOC105052110	AT1G62660
42	Invertase, beta-fructofuranosidase, insoluble isoenzyme 4	Synthesis	EgcWINV4	2	22223773-22228792	LOC105038260	AT1G12240
43	UTP--glucose-1-phosphate uridylyltransferase	Synthesis	EgUGP1	12	21117744-21128601	LOC105055260	AT3G03250
44	Hexokinase 2	Synthesis	EgHK2.1	1	12570216-12580885	LOC105040386	AT2G19860
45	Hexokinase 2	Synthesis	EgHK2.2	3	7087346-7097240	LOC105040520	AT2G19860
46	Hexokinase 2	Synthesis	EgHK2.3	4	53340928-53346372	LOC105044145	AT2G19860
47	Hexokinase 2	Synthesis	EgHK2.4	6	35270204-35283388	LOC105047281	AT2G19860
48	Hexokinase 2	Synthesis	EgHK2.5	Un	3653924-3674643	LOC105060091	AT2G19860
49	Hexokinase 3	Synthesis	EgHK3.1	5	27601549-27641956	LOC105045358	AT1G50460
50	Hexokinase 3	Synthesis	EgHK3.2	14	11855216-11867187	LOC105057669	AT1G50460
51	Fructokinase 1	Synthesis	EgFRK1.1	9	25179006-25181211	LOC105051457	AT2G31390
52	Fructokinase 1	Synthesis	EgFRK1.2	2	1481183-1483075	LOC105035371	AT2G31390
53	Fructokinase-like 1	Synthesis	EgFRK-like 1	8	34271475-34273596	LOC105050565	AT3G54090
54	Fructokinase 2	Synthesis	EgFRK2.1	8	16278303-16282759	LOC105049809	AT1G06030
55	Fructokinase 2	Synthesis	EgFRK2.2	2	2970589-2977588	LOC105034592	AT1G06030

56	Fructokinase-like 2	Synthesis	EgFRK-like 2	1	43609853-43626926	LOC105057042	AT1G69200
57	Fructokinase 6	Synthesis	EgFRK6.1	10	27143903-27162315	LOC105053244	AT1G66430
58	Fructokinase 6	Synthesis	EgFRK6.2	4	36920590-36926241	LOC114914109	AT1G66430
59	Fructokinase 7	Synthesis	EgFRK7	4	36916734-36920861	LOC105043531	AT5G51830
60	α-glucan phosphorylase 1	Degradation	EgPHO1	1	45199566-45212347	LOC105058113	AT3G29320
61	α-glucan phosphorylase 1	Degradation	EgPHO1b	5	50395879-50405236	LOC105046284	
62	α-glucan phosphorylase 2	Degradation	EgPHO2	1	17414711-17439100	LOC105043558	AT3G46970
63	α-glucan water dikinase 1	Degradation	EgGWD1	15	23704549-23728459	LOC105058794	AT1G10760
64	α-glucan water dikinase 2	Degradation	EgGWD2	10	25554098-25588529	LOC105053333	AT4G24450
65	Pjosphoglucan water dikinase	Degradation	EgPWD	Un	199719-290190	LOC105034176	AT5G26570
66	Early starvation 1	Degradation	EgESV1	4	9170942-9208651	LOC105042638	AT1G42430
67	Like Early starvation	Degradation	EgLESV	3	48353523-48361667	LOC105042207	AT3G55760
68	Phosphoglucan phosphatase	Degradation	EgSEX4	4	26772838-26806987	LOC105042928	AT3G52180
69	Phosphoglucan phosphatase	Degradation	EgLSF1	1	20080922-20100042	LOC105048130	AT3G10940
70	Phosphoglucan phosphatase	Degradation	EgLSF2	10	6117341-6126591	LOC105052271	AT3G01510
71	Limit dextrinase	Degradation	EgLDA (PU1)	4	32749208-32819925	LOC105043173	AT5G04360
72	4-α-glucanotransferase, Disproportionating Enzyme	Degradation	EgDPE1	3	27545735-27563049	LOC105041676	AT5G64860
73	4-α-glucanotransferase, Disproportionating Enzyme	Degradation	EgDPE2	14	11115576-11135195	LOC105057692	AT2G40840
74	α-1,6-glucosidase, Starch debranching enzyme	Degradation	EgISA1	7	42123581-42134692	LOC105049284	AT2G39930
75	α-1,6-glucosidase, Starch debranching enzyme	Degradation	EgISA2	14	16071499-16074389	LOC105057759	AT1G03310
76	α-1,6-glucosidase, Starch debranching enzyme	Degradation	EgISA3	Un	1845695-1876961	LOC105060617	AT4G09020
77	Exo-amylase, β-amylase 1	Degradation	EgBAM1	4	34729250-34732567	LOC105043204	AT3G23920
78	Exo-amylase, β-amylase 2	Degradation	EgBAM2	12	13114474-13120426	LOC105054866	AT4G00490
79	Exo-amylase, β-amylase 3	Degradation	EgBAM3	4	47365596-47370500	LOC105043800	AT4G17090
80	Exo-amylase, β-amylase 4	Degradation	EgBAM4	1	28379792-28384884	LOC105052391	AT5G55700
81	Exo-amylase, β-amylase 7	Degradation	EgBAM7	3	15198038-15203728	LOC105040934	AT2G45880
82	Exo-amylase, β-amylase 8	Degradation	EgBAM8	Un	519-4389	LOC105036549	AT5G45300
83	Exo-amylase, β-amylase 8-like	Degradation	EgBAM8-like	16	15504990-15509883	LOC105059706	
84	Exo-amylase, β-amylase 9	Degradation	EgBAM9	14	15296940-15298308	LOC109506615	AT5G18670
85	Endo-amylase, α-amylase 1	Degradation	EgAMY1	Un	1266914-1269728	LOC105060892	AT4G25000
86	Endo-amylase, α-amylase 2	Degradation	EgAMY2	4	7642412-7650671	LOC105042584	AT1G76130

87	Endo-amylase, α-amylase 3	Degradation	EgAMY3	6	42701527-42729908	LOC105047709	AT1G69830
88	α-glucosidase	Degradation	EgAGL	Un	2041534-2045204	LOC105059813	AT5G11720
89	Sugar transporter 1	Transporter	EgMST1	13	26849080-26853372	LOC105056823	AT1G11260
90	Sugar transporter 3	Transporter	EgMST3	1	61300079-61304175	LOC105032198	AT5G61520
91	Sugar transporter 4	Transporter	EgMST4.1	Un	767396-774089	LOC105032465	AT3G19930
92	Sugar transporter 4	Transporter	EgMST4.2	1	38038875-38042631	LOC105055157	AT3G19930
93	Sugar transporter 6	Transporter	EgMST6.1	Un	73804-77960	LOC105032731	AT3G05960
94	Sugar transporter 6	Transporter	EgMST6.2	4	19879656-19882112	LOC105042783	AT3G05960
95	Sugar transporter 6	Transporter	EgMST6.3	Un	1015113-1017493	LOC105032981	AT3G05960
96	Sugar transporter 7	Transporter	EgMST7	Un	2279487-2284036	LOC105060237	AT4G02050
97	Sugar transporter 14	Transporter	EgMST14	6	36502534-36507764	LOC105047352	AT1G77210
98	ADPGlucose transporter	Transporter	EgBT1	3	343425-355497	LOC105040171	AT4G32400
99	Sucrose Transporter 1	Transporter	EgSUT1	3	28219853-28233003	LOC105041710	AT1G22710
100	Sucrose Transporter 2	Transporter	EgSUT2	13	2589571-2604030	LOC105055917	AT2G02860
101	Sucrose Transporter 4	Transporter	EgSUT4	12	23825476-23841797	LOC105055489	AT1G09960
102	ATP-ADP antiporter 1	Transporter	EgNTT1	6	43851301-43856761	LOC105047760	AT1G80300
103	ATP-ADP antiporter 2	Transporter	EgNTT2	5	43708096-43721742	LOC105045949	AT1G15500
104	glucose 6-phosphate/phosphate translocator 2	Transporter	EgGPT2.1	10	15387451-15392278	LOC105052637	AT1G61800
105	glucose 6-phosphate/phosphate translocator 2	Transporter	EgGPT2.2	1	30265116-30269032	LOC105051762	AT1G61800
106	phosphate/triose-phosphate translocator precursor 1	Transporter	EgTPT1.1	3	15452560-15461702	LOC105040952	AT5G46110
107	phosphate/triose-phosphate translocator precursor 1	Transporter	EgTPT1.2	Un	902848-921288	LOC105032235	AT5G46110
108	phosphate/phosphoenolpyruvate translocator precursor 1	Transporter	EgPPT1.1	6	28668050-28676248	LOC105047036	AT5G33320
109	phosphate/phosphoenolpyruvate translocator precursor 1	Transporter	EgPPT1.2	1	22811757-22818527	LOC105046355	AT5G33320
110	Maltose Transporter	Transporter	EgMEX1	11	2855852-2863997	LOC105053537	AT5G17520
111	Glucose Transporter pGlcT 1	Transporter	EgPGlcT1	2	43216376-43241760	LOC105039104	AT1G05030
112	Glucose Transporter pGlcT 2	Transporter	EgPGlcT2	3	13692240-13699822	LOC105040839	AT1G67300
113	Glucose Transporter pGlcT 4	Transporter	EgPGlcT4	7	13962818-13976135	LOC105048412	AT5G16150
