# Supplementary material for: Transcriptomic analysis of mesocarp tissue during fruit development of the oil palm revealed specific isozymes related to starch metabolism that control oil yield
Source: Front Plant Sci. 2023 Jul 24;14:1220237. doi: 10.3389/fpls.2023.1220237 (PMC10405827; doi:10.3389/fpls.2023.1220237)
Supplement: Supplementary file 9 [file DataSheet_9.pdf]

Table S2. List of NCBI database accession number and summary of sequencing reads after filtering.

No	Sample name	Tissue	Organism name	Biosample accession no	SRA accession no	Total Number of Reads	Total Bases (Gb)	GC Content %
1	High yield 22WAP	Mesocarp	*Elaeis guineensis*	SAMN30940208	SRR21644179	53270932	7.1	48.44
2	High yield 20WAP	Mesocarp	*Elaeis guineensis*	SAMN30940207	SRR21644180	51705270	7.7	47.78
3	High yield 16WAP	Mesocarp	*Elaeis guineensis*	SAMN30940206	SRR21644181	48998078	7.3	47.51
4	High yield 12WAP	Mesocarp	*Elaeis guineensis*	SAMN30940205	SRR21644182	45026608	6.7	48.54
5	High yield 8WAP	Mesocarp	*Elaeis guineensis*	SAMN30940204	SRR21644183	41263926	6.1	48.63
6	High yield 4WAP	Mesocarp	*Elaeis guineensis*	SAMN30940203	SRR21644184	48181746	6.6	47.91
7	Medium yield 22WAP	Mesocarp	*Elaeis guineensis*	SAMN30940202	SRR21644185	50941938	6.9	47.28
8	Medium yield 20WAP	Mesocarp	*Elaeis guineensis*	SAMN30940201	SRR21644186	54385100	8.1	47.81
9	Medium yield 16WAP	Mesocarp	*Elaeis guineensis*	SAMN30940200	SRR21644171	53180460	7.9	48.08
10	Medium yield 12WAP	Mesocarp	*Elaeis guineensis*	SAMN30940199	SRR21644172	54485808	8.1	49.02
11	Medium yield 8WAP	Mesocarp	*Elaeis guineensis*	SAMN30940198	SRR21644173	44107804	6.6	48.51
12	Medium yield 4WAP	Mesocarp	*Elaeis guineensis*	SAMN30940197	SRR21644174	51332122	7.1	47.32
13	Low yield 22WAP	Mesocarp	*Elaeis guineensis*	SAMN30940196	SRR21644175	51937118	7.1	46.77
14	Low yield 20WAP	Mesocarp	*Elaeis guineensis*	SAMN30940195	SRR21644176	44285520	6.6	49.52
15	Low yield 16WAP	Mesocarp	*Elaeis guineensis*	SAMN30940194	SRR21644177	50582890	7.6	49.33
16	Low yield 12WAP	Mesocarp	*Elaeis guineensis*	SAMN30940193	SRR21644178	55212786	8.2	50.32
17	Low yield 8WAP	Mesocarp	*Elaeis guineensis*	SAMN30940192	SRR21644187	46375880	6.9	48.09
18	Low yield 4WAP	Mesocarp	*Elaeis guineensis*	SAMN30940191	SRR21644188	49044678	6.7	47.69
