# Supplementary material for: Transcriptomic analysis of mesocarp tissue during fruit development of the oil palm revealed specific isozymes related to starch metabolism that control oil yield
Source: Front Plant Sci. 2023 Jul 24;14:1220237. doi: 10.3389/fpls.2023.1220237 (PMC10405827; doi:10.3389/fpls.2023.1220237)
Supplement: Supplementary file 10 [file DataSheet_10.pdf]

Table S3. List of primers for qRT-PCR validation.

Locus	Gene	Primer	Name	Sequence (5'-3')	Product (Bp)
LOC105047182	EgAPL1	Forward	EgAPL1-F	GCAGAGGGTAAGGTGCCAAT	137
		Reverse	EgAPL1-R	TCACACGGCCTATCAGCTTC	
LOC105040918	EgSS1	Forward	EgSS1-F	CCAGATTCGAGCCATGTGGT	141
		Reverse	EgSS1-R	CCGTCCCCTGACCACTATCT	
LOC105043800	EgBAM3	Forward	EgBAM3-F	AGAGCAACGGAACTTGGAGG	155
		Reverse	EgBAM3-R	AGGGAACTGCTTGTAGTGCC	
LOC105047709	EgAMY3	Forward	EgAMY3-F	TGGGGAGTCTGCAGGGATAA	133
		Reverse	EgAMY3-R	CCCGACTACCAAGGCCATTT	
LOC105041710	EgSUT1	Forward	EgSUT1-F	CTCTCGGATTCCCCCTAGCT	147
		Reverse	EgSUT1-R	CACCGAGAGCTACGATCACC	
LOC105040520	EgHK2.2	Forward	EgHK2-F	CGACAGCAAGCTCAAGATGC	137
		Reverse	EgHK2-R	GCCTTTCCTTTCCACCCAGT	
LOC105035371	EgFRK1.2	Forward	EgFRK1-F	ATGAGGTTGCCCTGAAGCTC	140
		Reverse	EgFRK1-R	GCCTGTCGTATCCACCTGTT	
LOC105038179	EgPK	Forward	EgPK-F	CGACATCCCGCTGAGTCTAC	120
		Reverse	EgPK-R	GACCTTCTGGCAGACGACAA	
LOC105038005	EgACT3	Forward	EgACT3-F	TTCCAGCAGGTACGATGTGG	142
		Reverse	EgACT3-R	CCGGCAACCCTACATGACTT	
