# Supplementary material for: Transcriptomic analysis of mesocarp tissue during fruit development of the oil palm revealed specific isozymes related to starch metabolism that control oil yield
Source: Front Plant Sci. 2023 Jul 24;14:1220237. doi: 10.3389/fpls.2023.1220237 (PMC10405827; doi:10.3389/fpls.2023.1220237)
Supplement: Supplementary file 11 [file DataSheet_11.pdf]

Table S5. List of genes involved in glycolysis in oil palm.

No	Function	Pathway	Gene	Chr	Position	Oil Palm Locus
1	Phosphofructokinase 2	Glycolysis	EgPFK2.1	Un	2595498-2599419	LOC105060774
2	Phosphofructokinase 2	Glycolysis	EgPFK2.2	8	20796383-20800182	LOC105049935
3	Phosphofructokinase 3	Glycolysis	EgPFK3.1	1	13131876-13141905	LOC105040103
4	Phosphofructokinase 3	Glycolysis	EgPFK3.2	3	6770088-6777204	LOC105040501
5	Phosphofructokinase 3	Glycolysis	EgPFK3.3	7	4221116-4228601	LOC105048019
6	Phosphofructokinase 3	Glycolysis	EgPFK3.4	6	35471198-35484751	LOC105047293
7	Phosphofructokinase 5	Glycolysis	EgPFK5.1	3	28622022-28635737	LOC105041727
8	Phosphofructokinase 5	Glycolysis	EgPFK5.2	3	24809569-24823740	LOC105041408
9	Phosphofructokinase 5	Glycolysis	EgPFK5.3	15	15599864-15614455	LOC105058322
10	Fructose-1 6-bisphosphate aldolase 1	Glycolysis	EgFBA1.1	10	22002485-22006520	LOC105052993
11	Fructose-1 6-bisphosphate aldolase 1	Glycolysis	EgFBA1.2	1	49568840-49573291	LOC105059022
12	Fructose-1 6-bisphosphate aldolase 1	Glycolysis	EgFBA1.3	7	12397612-12403435	LOC105048474
13	Fructose-1 6-bisphosphate aldolase 1	Glycolysis	EgFBA1.4	8	5259677-5265049	LOC105049575
14	Fructose-1 6-bisphosphate aldolase 1	Glycolysis	EgFBA1.5	2	21292073-21298171	LOC105038211
15	Fructose-1 6-bisphosphate aldolase 2, chloroplastic	Glycolysis	EgFBA2.1	9	28994281-28998020	LOC105051883
16	Fructose-1 6-bisphosphate aldolase 2, chloroplastic	Glycolysis	EgFBA2.2	2	24879552-24882967	LOC105038393
17	Fructose-1 6-bisphosphate aldolase 2, chloroplastic	Glycolysis	EgFBA2.3	3	19104216-19109605	LOC105041129
18	Fructose-1 6-bisphosphate aldolase 2, chloroplastic	Glycolysis	EgFBA2.4	4	42500996-42503166	LOC105043516
19	Fructose-1 6-bisphosphate aldolase 5	Glycolysis	EgFBA5.1	8	34586864-34589262	LOC105050625
20	Fructose-1 6-bisphosphate aldolase 5	Glycolysis	EgFBA5.2	Un	532179-535245	LOC105060505
21	Triose phosphate isomerase	Glycolysis	EgTPI1.1	5	48610041-48620337	LOC105045658
22	Triose phosphate isomerase	Glycolysis	EgTPI1.2	14	1691696-1699281	LOC105056934
23	Triose phosphate isomerase	Glycolysis	EgTPI1.3	5	1321644-1329452	LOC105044400
24	Triose phosphate isomerase, chloroplastic	Glycolysis	EgTPI2.1	12	17232744-17241128	LOC105055051
25	Triose phosphate isomerase, chloroplastic	Glycolysis	EgTPI2.2	16	19612903-19624462	LOC105059611
26	Glyceraldehyde-3-phosphate dehydrogenase 1	Glycolysis	EgG3PDH1.1	Un	138602-142971	LOC105034336
27	Glyceraldehyde-3-phosphate dehydrogenase 1	Glycolysis	EgG3PDH1.2	Un	23569-24788	LOC105036324

28	Glyceraldehyde-3-phosphate dehydrogenase 1	Glycolysis	EgG3PDH1.3	Un	21286-22483	LOC105036331
29	Glyceraldehyde-3-phosphate dehydrogenase 2	Glycolysis	EgG3PDH2.1	9	21230743-21236873	LOC105051363
30	Glyceraldehyde-3-phosphate dehydrogenase 2	Glycolysis	EgG3PDH2.2	Un	40-1314	LOC105037818
31	Glyceraldehyde-3-phosphate dehydrogenase 1, chloroplastic	Glycolysis	EgGAPCP1	Un	4126227-4135720	LOC105059882
32	Glyceraldehyde-3-phosphate dehydrogenase 2, chloroplastic	Glycolysis	EgGAPCP2	2	55637323-55645779	LOC105039685
33	Phosphoglycerate kinase 1	Glycolysis	EgPGK1.1	1	39791248-39795367	LOC105056168
34	Phosphoglycerate kinase 1	Glycolysis	EgPGK1.2	Un	151988-184808	LOC105033802
35	Phosphoglycerate kinase 3	Glycolysis	EgPGK3	Un	3789742-3794456	LOC105059872
36	Phosphoglycerate kinase, chloroplastic	Glycolysis	EgpPGK	6	2957610-2960881	LOC105046543
37	Phosphoglycerate mutase 1	Glycolysis	EgPGAM1.1	10	8440907-8449744	LOC105052340
38	Phosphoglycerate mutase 1	Glycolysis	EgPGAM1.2	13	27759088-27767081	LOC105056873
39	Phosphoglycerate mutase 1	Glycolysis	EgPGAM1.3	15	16943025-16951676	LOC105058395
40	Phosphoglycerate mutase 1	Glycolysis	EgPGAM1.3	5	8744695-8754497	LOC105044879
41	Phosphoglycerate mutase 1	Glycolysis	EgPGAM1.4	5	41729301-41739929	LOC105046041
42	Phosphoglycerate mutase-like	Glycolysis	EgPGAM-like	14	5288787-5307541	LOC105057242
43	Phosphoglycerate mutase-like 4	Glycolysis	EgPGAM-like 4	Un	46424-61530	LOC105034420
44	Enolase 1	Glycolysis	EgENO1	Un	226246-232255	LOC105034448
45	Enolase 2	Glycolysis	EgENO2	11	4302397-4309920	LOC105053561
46	Enolase 3	Glycolysis	EgENO3	8	34184655-34195395	LOC105050564
47	Enolase, chloroplastic	Glycolysis	EgpENO	2	45765178-45769230	LOC105039219
48	Pyruvate kinase 1	Glycolysis	EgPK1.1	2	20142744-20154355	LOC105038179
49	Pyruvate kinase 1	Glycolysis	EgPK1.2	9	31745910-31755594	LOC105051730
50	Pyruvate kinase 1	Glycolysis	EgPK1.3	12	23584887-23597337	LOC105055472
51	Pyruvate kinase 1	Glycolysis	EgPK1.4	14	5819611-5823285	LOC105057280
52	Pyruvate kinase 1	Glycolysis	EgPK1.5	Un	50528-204207	LOC105034683
53	Pyruvate kinase 1	Glycolysis	EgPK1.6	Un	827-3953	LOC105035292
54	Pyruvate kinase 2	Glycolysis	EgPK2.1	6	39759818-39768421	LOC105047533
55	Pyruvate kinase 2	Glycolysis	EgPK2.2	16	3490440-3499010	LOC105058982
56	Pyruvate kinase 2	Glycolysis	EgPK2.3	5	9864581-9868306	LOC105044926
57	Pyruvate kinase 3	Glycolysis	EgPK3	4	3026097-3033699	LOC105042489
58	Pyruvate kinase 4	Glycolysis	EgPK4.1	7	7483872-7492014	LOC105048201
59	Pyruvate kinase 4	Glycolysis	EgPK4.2	3	11273494-11279022	LOC105040705

60	Pyruvate kinase 5	Glycolysis	EgPK5	1	50297714-50302868	LOC105059219
61	Pyruvate kinase, isozyme G , chloroplastic	Glycolysis	EgPKG.1	12	16043659-16052105	LOC105055006
62	Pyruvate kinase, isozyme G , chloroplastic	Glycolysis	EgPKG.2	Un	162-1574	LOC105037122
63	Pyruvate kinase, isozyme G , chloroplastic	Glycolysis	EgPKG.3	4	48180715-48191332	LOC105043843
64	Pyruvate kinase, isozyme A , chloroplastic	Glycolysis	EgPKA.1	6	3477114-3489928	LOC105046576
65	Pyruvate kinase, isozyme A , chloroplastic	Glycolysis	EgPKA.2	1	40816119-40825006	LOC105055684
66	Pyruvate kinase, isozyme A , chloroplastic	Glycolysis	EgPKA.3	Un	3572911-3579106	LOC105060087
67	Pyruvate kinase - like	Glycolysis	EgPK-like	5	40400284-40403662	LOC105046216
