# Supplementary material for: Transcriptomic analysis of mesocarp tissue during fruit development of the oil palm revealed specific isozymes related to starch metabolism that control oil yield
Source: Front Plant Sci. 2023 Jul 24;14:1220237. doi: 10.3389/fpls.2023.1220237 (PMC10405827; doi:10.3389/fpls.2023.1220237)
Supplement: Supplementary file 2 [file DataSheet_2.pdf]

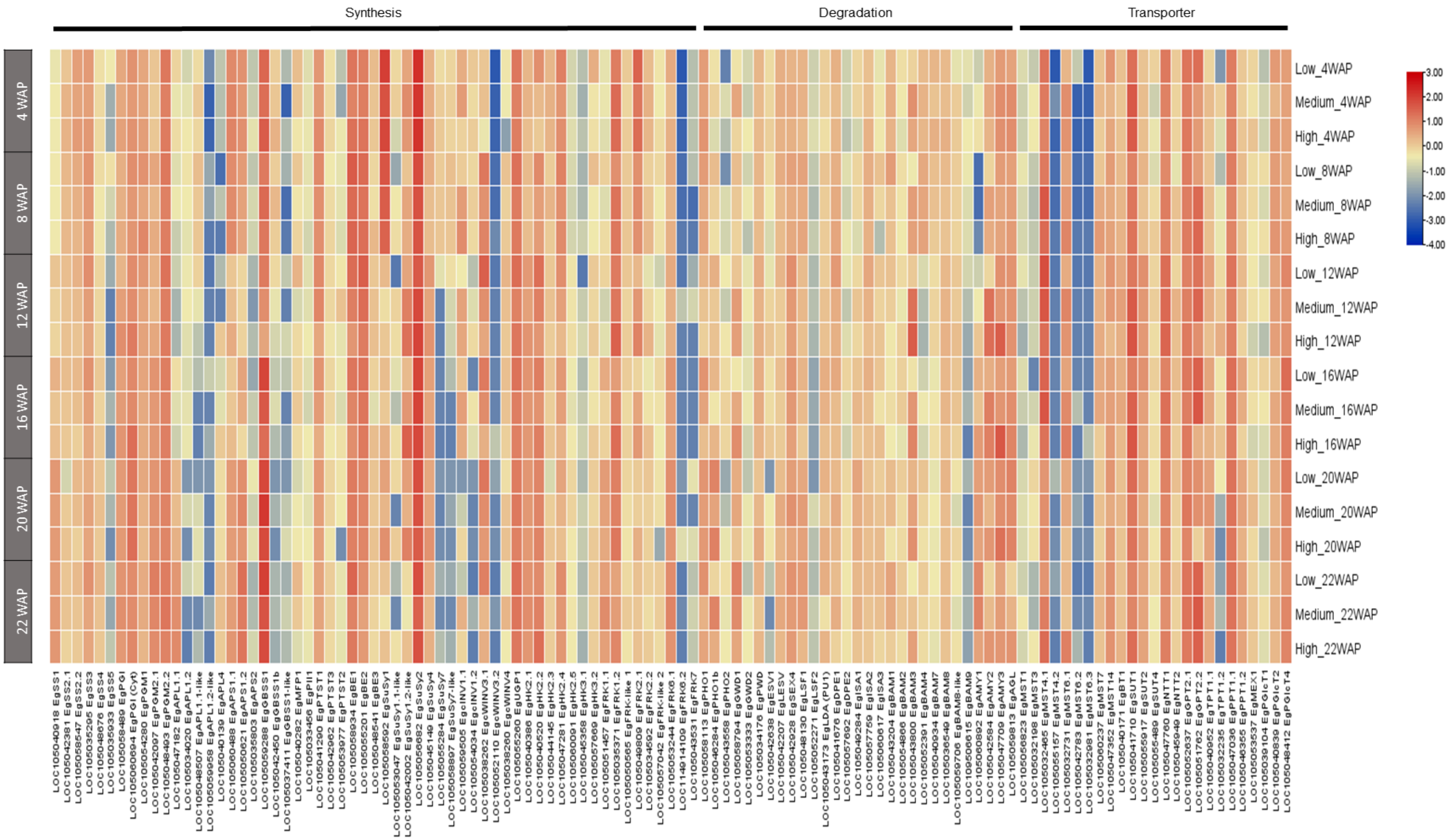

Figure S2. Heatmap analysis of starch-related metabolism genes during fruit development. The genes related to synthesis, degradation, and transporter are indicated at the top of the figure. Comparison of gene expression with altered oil yield at different developmental stages. Color corresponds to per-gene z-score that is computed from normalized values of log2 (CPM+1).
